# Study of the antioxidant and anti-pancreatic cancer activities of *Anchusa strigosa* aqueous extracts obtained by maceration and ultrasonic extraction techniques

**DOI:** 10.3389/fphar.2023.1201969

**Published:** 2023-08-01

**Authors:** Ziad Chebaro, Rola Abdallah, Adnan Badran, Kamar Hamade, Akram Hijazi, Marc Maresca, Joelle Edward Mesmar, Elias Baydoun

**Affiliations:** ^1^ Platforme de Recherche et D’analyse en Sciences de L’environnement (EDST-PRASE), Beirut, Lebanon; ^2^ Department of Biology, American University of Beirut, Beirut, Lebanon; ^3^ Department of Nutrition, University of Petra, Amman, Jordan; ^4^ UMRT INRE 1158 BioEcoAgro, Laboratorie BIOPI, University of Picardie Jules Verne, Amiens, France; ^5^ Aix-Marseille Univ, CNRS, Centrale Marseille, iSM2, Marseille, France

**Keywords:** *Anchusa strigosa*, pancreatic cancer, herbal medicine, bioactive compounds, conventional extraction, ultrasound-assisted extraction, apoptosis, COX-2

## Abstract

Pancreatic cancer is a highly aggressive malignancy and a leading cause of cancer-related deaths worldwide. Moreover, the incidence and mortality rates for pancreatic cancer are projected to keep increasing. A major challenge in the treatment of pancreatic cancer is the lack of effective screening approaches, which contributes to its poor prognosis, indicating the need for new treatment regimens and alternative therapies, such as herbal medicine. The medicinal plant *A. strigosa*, which is widely distributed in the Eastern Mediterranean region, is a short prickly plant from the Boraginaceae family that has been widely used in traditional medicine for treating various diseases. Nevertheless, its effect on human pancreatic cancer remains poorly investigated. In the present study, we screened the phytochemical content of *Anchusa strigosa* aqueous extracts obtained by maceration and ultrasound-assisted methods (ASM and ASU, respectively) and evaluated their antioxidant effects. We also investigated their anticancer effects and possible underlying mechanisms. The results show that both extracts were rich in bioactive molecules, with slight differences in their composition. Both extracts exhibited remarkable antioxidant potential and potent radical-scavenging activity *in vitro*. Additionally, non-cytotoxic concentrations of both extracts attenuated cell proliferation in a time- and concentration-dependent manner, which was associated with a decrease in the proliferation marker Ki67 and an induction of the intrinsic apoptotic pathway. Furthermore, the extracts increased the aggregation of pancreatic cancer cells and reduced their migratory potential, with a concomitant downregulation of integrin β1. Finally, we showed that the ASM extract caused a significant decrease in the levels of COX-2, an enzyme that has been linked to inflammation, carcinogenesis, tumor progression, and metastasis. Taken together, our findings provide evidence that *A. strigosa* extracts, particularly the extract obtained using the maceration method, have a potential anticancer effect and may represent a new resource for the design of novel drugs against pancreatic cancer.

## 1 Introduction

Cancer is the second leading cause of death worldwide, after heart disease. New cases have been growing by 1% each year since the late 1990s, affecting both men and women, and reaching over 495,000 new cases in 2020 (Cancer, Net, 2023; World Cancer Research Fund International, 2023). Numbers are expected to grow, due to the aging and growth of the global population, from 18.1 million new cases in 2018 to around 29.4 million cases in 2040, equivalent to a predicted 60% increase (The Cancer Atlas, 2018). Moreover, experts estimate that it will be the top cause of death worldwide in this century (Saez, 2018). Although our understanding of cancer has evolved throughout the years, cancer continues to wreck lives, bringing sorrow and pain. The burden of cancer on society and health systems is huge. Of particular concern is pancreatic cancer, which is considered one of the most aggressive and lethal malignancies because of its poor prognosis. Pancreatic cancer is asymptomatic and silent for a long time and, by the time it becomes symptomatic, it is often incurable. At the time of diagnosis, only 10%–20% can have surgery, which until now has been the best chance for cure and the only curative option (Lambert et al., 2019). However, local or distant relapse remains a major obstacle. If the tumor is unresectable, treatment consists of chemotherapy, sometimes in combination with radiation, with often inconsistent results, poor outcomes, and controversial benefits (Hajatdoost et al., 2018). In other words, pancreatic cancer accounts for almost as many deaths as cases. Moreover, the incidence of pancreatic cancer is on the rise, prompting an urgent need to improve survival rates (Rahib et al., 2014; Rawla et al., 2019). As such, researchers have been looking for alternative treatments for pancreatic cancer, with herbal medicine increasingly gaining interest and popularity due its lower costs and fewer side effects. So far, natural products and herbal formulas have been shown to be safe and effective options with good therapeutic effects against cancer in general and pancreatic cancer in particular (Saif, 2008; Zhang et al., 2022).

**FIGURE 1:**
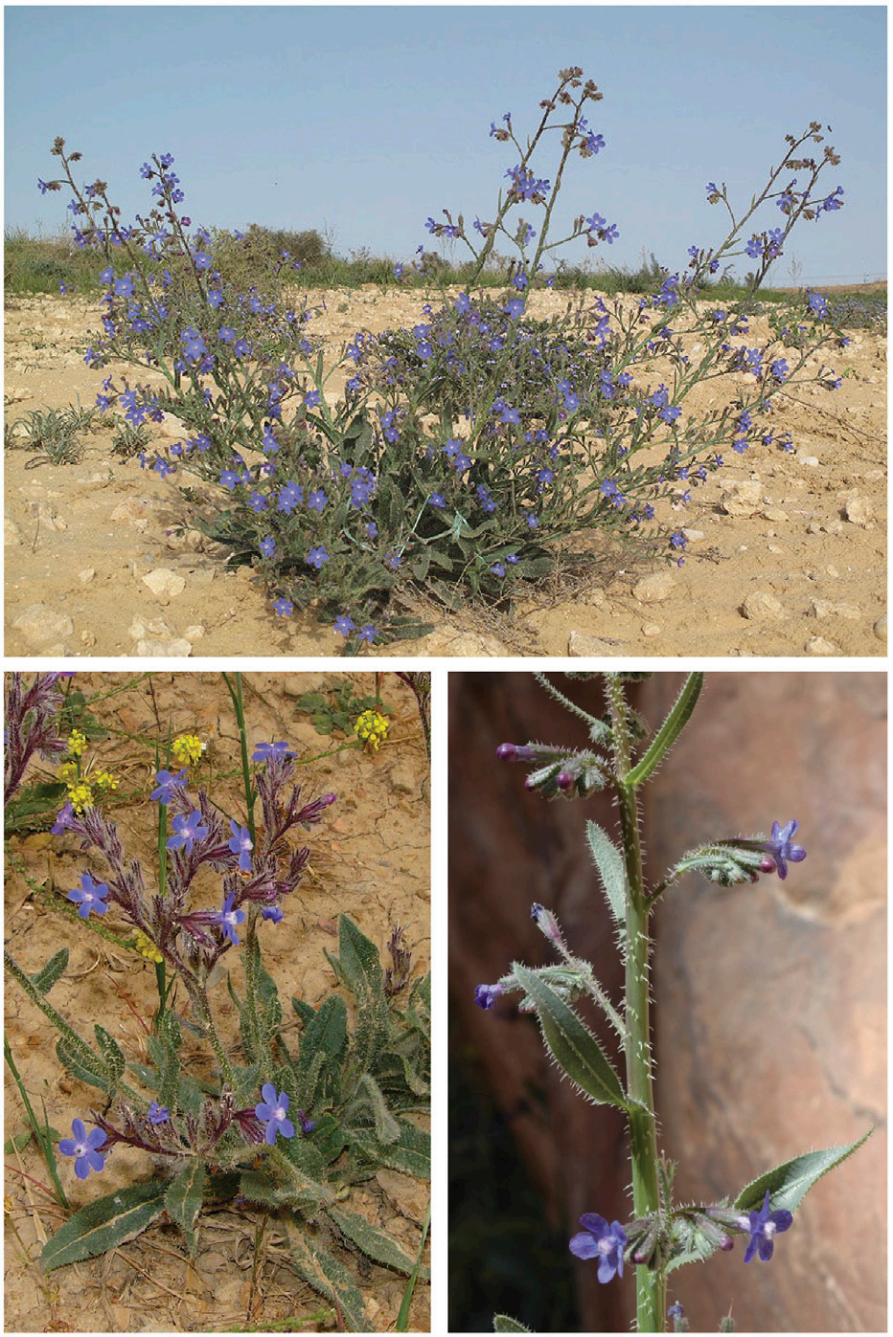
*Anchusa strigosa* plant. Images obtained from https://powo.science.kew.org/taxon/urn:lsid:ipni.org:names:113367-1 (accessed on 25 March 2023).

*Anchusa strigosa*, commonly known as prickly alkanet or “Lisan Al-thawr” in Arabic, is a perennial weed from the family Boraginaceae, that, is widely distributed in the Eastern Mediterranean region and native to Lebanon, Syria, Jordan, Iraq, and Iran. It grows on roadsides and has the ability to adapt to a wide range of habitats, from Mediterranean woods and steppe vegetation to true deserts (Tohme and Tohmé, 2014). The plant consists of a basal rosette of leaves and an inflorescence of pure white or cobalt blue flowers borne on a stem that can reach up to 1 m in length (Figure 1). It is traditionally used in folk medicine for analgesic and sedative treatments, and to relieve pain (Al-douri 2000; Yeşil and Inal, 2021). Extracts from *A. strigosa* have been shown to be effective in the alleviation or treatment of several diseases (Yarmolinsky et al., 2022). It has been reported to have anti-bacterial (Abutbul et al., 2005; Al-Salihi et al., 2007; Al-Salihi et al., 2009), anti-fungal (Ali Shtayeh and Abu Ghdeib, 1999), anti-ulcer (Disi et al., 1998; Abbas et al., 2009), anti-arthritic (Alallan et al., 2018), and antioxidant (Alali et al., 2007; Dibas et al., 2017; Al-Khateeb et al., 2019) activities. The anticancer activity of *A. strigosa* against several cancer cell lines from breast and colorectal carcinomas has also been investigated (Dibas et al., 2017; Al-Khatib et al., 2021). This wide range of activities is attributed to the fact that a variety of bioactive compounds and phytochemicals, namely, polyphenols, flavonoids, terpenoids, saponins, alkaloids, glycosides, steroids, and sterols, are bountiful in *A. strigosa* extracts (Alallan et al., 2018; Ghalib and Kadhim, 2021). These studies suggest that *A. strigosa* may have beneficial anticancer properties, which promoted us to investigate its effect in the context of pancreatic cancer.

The extraction of plant material is a crucial first step in the preparation of plant samples rich in active biomolecules (Stéphane et al., 2021). Selection of appropriate extraction methods needs meticulous evaluation in order to ensure that therapeutic phytochemicals are preserved and stable.

In this study, the phytochemical composition and antioxidant activity of aqueous extracts of *A. strigosa* were evaluated using the traditional maceration technique (ASM) and the more modern ultrasound-assisted method (ASU). Moreover, the types of compounds in the extracts of *A. strigosa* leaves were also investigated. Finally, their effect on the malignant phenotype of the human pancreatic ductal adenocarcinoma cells (Capan-2) was tested. We examined the effects of the ASM and ASU extracts on the proliferation, migration, adhesion, and aggregation of Capan-2 cells. In addition, the molecular and mechanistic basis of their action was also investigated.

## 2 Materials and Methods

### 2.1 Aqueous extracts of *A. strigosa*

Leaves of *A. strigosa* were collected from Akkar, Lebanon, at 500 m altitude in April 2022. The leaves were rinsed, air-dried in the dark at room temperature, and then placed in an oven at 40°C for 24 h. The dried leaves were then crushed and ground into fine powder. Aqueous crude extracts of *A. strigosa*, ASM and ASU, were obtained by maceration and ultrasound-assisted extraction, respectively.

#### 2.1.1 Maceration (ASM)

*A. strigosa* powder was resuspended in distilled water at a ratio of 1:10 (w/v) in the dark for 48 h, with constant shaking. The aqueous suspension was centrifuged at 2,500 rpm for 10 min and vacuum-filtered. The supernatant was then lyophilized, and the powder obtained was dissolved in sterile ultrapure water at 100 mg/mL and kept in the dark at 4°C.

#### 2.1.2 Ultrasound-assisted extraction (ASU)

*A. strigosa* powder was immersed in distilled water at a ratio of 1:10 (w/v) and placed in an ultrasound bath (Wiseclean Sonicator) at 40 kHz for 30 min at a temperature below 40°C. The process of separation, filtration, and lyophilization was similar to that described above for ASM.

### 2.2 Total flavonoid content (TFC)

The total flavonoid content (TFC) of *A. strigosa* was determined by the aluminum chloride colorimetric assay, as reported by Quettier-Deleu et al. (2000) with slight modification. Each extract was prepared at a concentration of 1 mg/mL of sterile ultrapure water and an aliquot (1 mL) was mixed with 1 mL of 2% methanolic aluminum chloride. The mixture was incubated for 30 min in the dark and at room temperature. The absorbance was measured at 415 nm, using rutin as standard. The TFC was expressed as mg rutin equivalents (RE) per Gram of extract (mg RE/g). Analysis of TFC was repeated three times and data are presented as mean values ±SEM.

### 2.3 Total phenolic content (TPC)

With slight modification, the total phenolic content (TPC) of *A. strigosa* extracts was evaluated using the Folin–Ciocalteu method (Phuyal et al., 2020). A stock of each *A. strigosa* extract was prepared at a concentration of 1 mg/mL. An aliquot (500 μL) of the extract was oxidized with 2.5 mL of 0.2 N Folin–Ciocalteu reagent for 5 min. Then the reaction was neutralized with 2 mL of 75 g/L sodium carbonate and incubated in the dark at 37°C for 1 h. The absorbance was measured at 760 nm, where gallic acid was used as a standard. The TPC was expressed as a percentage of total gallic acid equivalents per Gram of extract (mg GAE/g). Analysis of TPC was repeated three times and data are presented as mean values ±SEM.

### 2.4 Assay of antioxidant activity

The antioxidant activity of the extracts was determined using the free-radical-scavenging activity of α, α-diphenyl-β-picrylhydrazyl (DPPH), as previously described in Farhan et al. (2012), with some modification. Increasing concentrations of each extract were tested (100, 200, 400, 600, 800 and 1,000 μg/mL). 0.5 mL from each concentration was mixed with 0.5 mL of DPPH solution (0.5 mM in methanol) and 3 mL of methanol. The blank consisted of 0.5 mL of distilled water with 0.5 mL of DPPH, and 3 mL of methanol. Ascorbic acid was used as a standard. The samples were then kept in the dark and at room temperature for 30 min and the absorbance was measured at a wavelength of 517 nm using a Hitachi U-2900 UV-Vis spectrophotometer. The DPPH- scavenging activity of each concentration of the extract was calculated as a percentage inhibition using the following formula:

$$Percentage\;of\;inhibition\;\left(\%\right)=\frac{absorbance\;of\;control-absorbance\;of\;the\;extract}{absorbance\;of\;control}\times 100$$

### 2.5 Liquid chromatography-mass spectrophotometry

For HPLC-PDA-MS/MS analysis, the mass spectrometer (LC-MS 8050; Shimadzu, Japan) was coupled to a triple quadruple spectrometer with an ESI source. The samples underwent chromatographic separation using a Zorbax Eclipse XDB-C18 column (4.6 mm i.d. × 150 mm long, 3.5 µm particle size; Agilent, Santa Clara, CA, United States). Two mobile phases, water and acetonitrile (ACN), were used and the gradient applied from 5% to 60% ACN over 40 min at a flow rate of 1 mL/min. The extracts were injected via an autosampler (SIL-40C xs) controlled by LC solution software (Shimadzu, Japan). The ions were collected in the negative mode.

### 2.6 Cell culture

Capan-2 human pancreatic cancer cells [CLS (Cell Line Service), Eppelheim, Germany] were maintained in a humidified (37°C and 5% CO_2_) chamber in high-glucose DMEM (Dulbecco’s modified Eagle’s medium), supplemented with 10% fetal bovine serum (FBS) (both from Sigma-Aldrich, St. Louis, MO, United States) and 1% penicillin/streptomycin (Lonza, Switzerland).

### 2.7 Assay of cell viability

Capan-2 cells (5 × 10^3^) were seeded in 96-well plates and allowed to grow until they reached 30%–40% confluency. The cells were then treated with increasing concentrations of extract and incubated for a total period of 72 h. Cell viability was assessed by the reduction of 3-(4,5- dimethylthiazol-2-yl)-2,5-diphenyltetrazolium bromide (MTT; Sigma-Aldrich, St. Louis, MO, United States). Cell growth was determined as the proportional viability of the treated cells in comparison with the vehicle (sterile ultrapure water)-treated cells, the viability of which was assumed to be 100%. The assay was performed in triplicate and repeated three times. Data are presented as mean values ±SEM.

### 2.8 Analysis of apoptotic morphological changes

Morphological changes characteristic of apoptotic cells were observed using a phase-contrast inverted microscope. For this, cells were grown in 6-well plates in the presence or absence of different concentrations of ASM and ASU. Pictures were taken after 24 h at 4×, ×10, and ×20 magnifications.

Changes in nuclear morphology characteristic of apoptosis were determined by 4′, 6-diamidino-2-phenylindole, dihydrochloride (DAPI) staining. Capan-2 cells were grown in 12-well plates in the presence or absence of the indicated concentrations of ASM and ASU for 24 h. Then the cells were fixed with 4% formaldehyde, stained with DAPI (Cell Signaling #4083) according to the manufacturer’s instructions, and visualized by fluorescence microscopy.

### 2.9 Wound-healing assay

Capan-2 cells were grown in 12-well plates until confluent. A wound scratch was made through the confluent monolayer using a sterile 200-μL plastic pipette tip. The culture medium was then removed and the cells were washed twice with phosphate-buffered saline (PBS; Sigma-Aldrich, St. Louis, MO, United States) to remove the remaining cellular debris. Fresh medium in the presence or absence of the indicated concentrations of ASM and ASU was added, and cells were further incubated at 37°C. Photomicrographs were taken at 0 (baseline), 8, and 12 h time points using an inverted phase-contrast microscope (objective ×4). The wound width was expressed as the average ±SEM between the measurements taken at time zero and the corresponding time points. The assay was repeated three times and data are presented as mean ± SEM.

### 2.10 Aggregation assay

Cell aggregation was assessed by harvesting cells from confluent plates using 2 mM EDTA in calcium and magnesium-free (CMF)-PBS and aliquoting them onto fresh empty dishes, with or without treatment. Cells were incubated at 37°C with shaking for 4 h and then fixed with 1% formaldehyde. Pictures were taken using the Olympus IX 71 inverted microscope.

### 2.11 Analysis of western blots

Cells were washed twice with PBS, scraped, and lysed using 2% SDS, 60 mM Tris lysis buffer (pH 6.8). The cell lysates were centrifuged at 5,000 *g* for 10 min and the protein concentration of the supernatants was determined using the Bradford protein assay kit (Bio-Rad, Hercules, CA, United States). Aliquots of 25–30 μg were resolved by 10% sodium dodecyl sulfate-polyacrylamide gel electrophoresis and transferred to a polyvinylidene difluoride membrane (Immobilon PVDF; Bio-Rad, Hercules, CA, United States). The membrane was then blocked with 5% nonfat dry milk in TBST (TBS and 0.05% Tween) for 1 h at room temperature. Immunodetection was carried out using the specified primary antibodies. Horseradish peroxidase-conjugated anti-IgG was used as secondary antibody and immunoreactive bands were detected using the Clarity Western ECL substrate kit (Bio-Rad, Hercules, CA, United States), according to the manufacturer’s instructions. All primary and secondary antibodies were obtained from Cell Signaling (Cell Signaling Technology, Inc., Danvers, MA, United States: β-actin #3700, caspase-3 #9662, BCL-2 #15071, integrin β1 #9699, and COX2 #12282) and abcam (Ki67 ab92742). For quantification, experiments were repeated three times. Data are presented as mean values ±SEM.

### 2.12 Statistical analysis

Data were statistically evaluated using the Student’s t-test with GraphPad Prism version 5.0. For the comparison of more than two means, ANOVA was used: either a one-way ANOVA (with Dunnett’s post-hoc test) or a two-way ANOVA (with Tukey–Kramer’s post-hoc test). Data are presented as mean ± standard error of the mean (SEM). A *p*-value of less than 0.05 is considered as significant.

## 3 Results

### 3.1 Analysis of *A. strigosa* extracts by LC-MS

**FIGURE 2:**
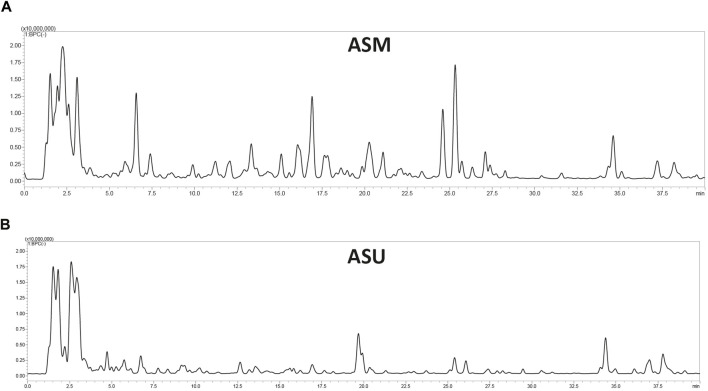
LC-MS profile of *A. strigosa* extracts obtained by maceration **(A)** and ultrasound-assisted **(B)** extraction methods.

Aqueous extracts obtained from *A. strigosa* leaves using the two extraction methods (maceration and ultrasonic extraction) were subjected to liquid chromatography analysis. The chromatographic peak profiles of ASM and ASU differed in the composition of their bioactive compounds, with the ASM extract showing more and higher peaks (Figure 2). Moreover, the study revealed the presence of 38 phytochemicals in ASM and 39 in ASU based on their MS, MS/MS spectral data, and retention times (Table 1). Rosmarinic acid was particularly abundant in the ASM extract.

**TABLE 1:** Annotated compounds from *A. strigosa* extracts using LC-MS/MS.

Rt (min)	[M-H]^-^	MS/MS	Compound name	ASM	ASU
0.91	191	109	Quinic acid	x	xx
1.51	193	103	Glucuronic acid	xx	xxx
1.65	191	111	Citric acid	xx	xxx
1.73	321	169	Digallic acid	xx	x
1.88	133	115	Malic acid	xx	x
1.90	115	115	Maleic acid	xx	xx
3.74	309	163	Hydroxycinnamic acid rhamnoside	xx	xxx
3.97	315	153	Dihydroxybenzoic acid glucoside	xx	xxx
4.03	153	109	Dihydroxybenzoic acid	x	x
4.46	169	124	Gallic acid	xx	xx
5.26	325	163	Hydroxycinnamic acid glucoside	x	x
5.59	359	197	Danshensu glucoside	xx	xx
5.69	515	341	Caffeoyl quinyl glucose		xx
6.14	329	153	Dihydroxybenzoic acid glucuronide	xxx	xx
6.41	521	197	Syringic acid diglucoside	xxx	xx
6.7	329	197	Syringic acid pentoside	xxx	xxx
7.29	153	109	Dihydroxybenzoic acid	xxx	xx
8.50	341	179	Caffeic acid glucoside	xx	xxx
8.68	353	191	Chlorogenic acid	xxx	xx
8.75	367	193	Feruloylquinic acid	xxx	xx
10.22	163	119	Coumaric acid	xx	x
10.63	355	193	Ferulic acid glucoside	xx	x
10.71	337	163	Coumaroylquinic acid	x	x
11.14	355	193	Ferulic acid *C*-glucoside	xx	x
12.52	353	191	Chlorogenic acid (isomer)	x	x
13.19	179	135	Caffeic acid	x	x
16.15	695	383	Apigenin di C- pentosyl-C-glucoside	xx	x
16.23	689	285	Kaempferol diglucoside Sulfate	xx	x
18.40	539	179	Caffeic acid derivative	xx	x
19.57	609	301	Quercetin rutinoside	x	xx
20.04	609	301	Quercetin rutinoside	x	xx
22.26	593	285	Kaempferol rutinoside	xx	x
24.49	359	179	Rosmarinic acid	xxx	x
25.19	537	295	Salvianolic acid J	xxx	xx
27.16	717	339	Salvianolic acid B	xx	x
37.67	395	197	Unknown	xx	xx
35.08	285	151	Kaempferol	x	x
36.95	327	171	Hydroxyicosanoic acid	xx	xx
38.27	327	197	Hydroxyicosanoic acid	xx	xx

Rt, retention time; X, minor; XX, moderate; XXX, major.

This indicates that the ASM extract contains more secondary bioactive metabolites. According to our results, the maceration method could be selected as being more effective for the extraction of bioactive compounds from *A. strigosa* than the ultrasound-assisted method.

### 3.2 The phenolic and flavonoid contents of *A. strigosa* extracts

Polyphenolic compounds are a large family of secondary metabolites that account for most of the antioxidant properties of plants. Our results show that the highest TPC and TFC contents were recorded in the ASM extract with 284.79 ± 12.4 mg GAE/g and 27.93 ± 0.39 mg RE/g extract, respectively, compared to 169.06 ± 7.11 mg GAE/g and 25.20 ± 0.21 mg RE/g extract, respectively, for ASU (Table 2). This is in accordance with the LC-MS data, which clearly highlighted the differences in the composition of the extracts and emphasized their differential abundance with regards to polyphenols.

**TABLE 2:** Total phenolic and flavonoid contents in *A. strigosa* crude extracts.

Parameters	ASM	ASU
**TPC** (mg GAE/g dry weight)	284.79 ± 12.38	169.06 ± 7.11
**TFC** (mg RE/g dry weight)	27.93 ± 0.39	25.20 ± 0.21

### 3.3 The antioxidant potential of *A. strigosa* extracts

Antioxidants are molecules that can prevent and decrease the oxidation of other molecules, reducing their capacity to damage by reacting with free radicals. Referred to as free-radical scavengers, antioxidant molecules can delay or inhibit oxidative stress or cellular damage.

**FIGURE 3:**
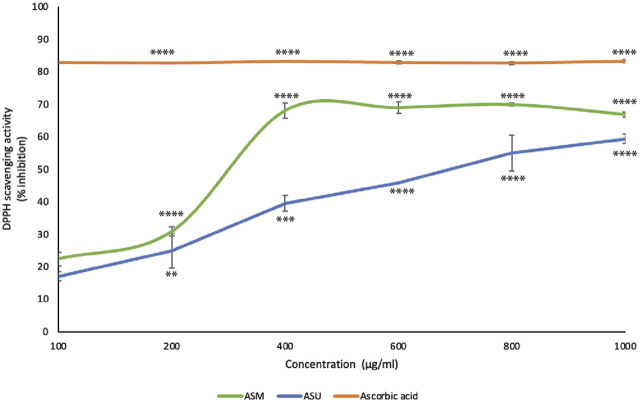
*A. strigosa* extracts have remarkable antioxidant potential. The antioxidant activity of the indicated concentrations of ASM and ASM was measured by the 2,2-di-phenyl-1-picrylhydrazyl (DPPH) assay for radical-scavenging capacity assay as described in Materials and Methods. Data represent the means ± SEM of three independent experiments (**p < 0.005, ***p < 0.001, ****p < 0.0001).

In the present study, both ASM and ASU extracts showed concentration-dependent free-radical scavenging activity (Figure 3). More specifically, ASM exhibited a stronger reducing power than ASU. These results correlate with the TPC, TFC, and LC-MS data, confirming that the greater the concentration of phenolics and flavonoids, the greater the antioxidant ability of the extract.

### 3.4 ASM and ASU inhibit the proliferation of Capan-2 pancreatic cancer cells

**FIGURE 4:**
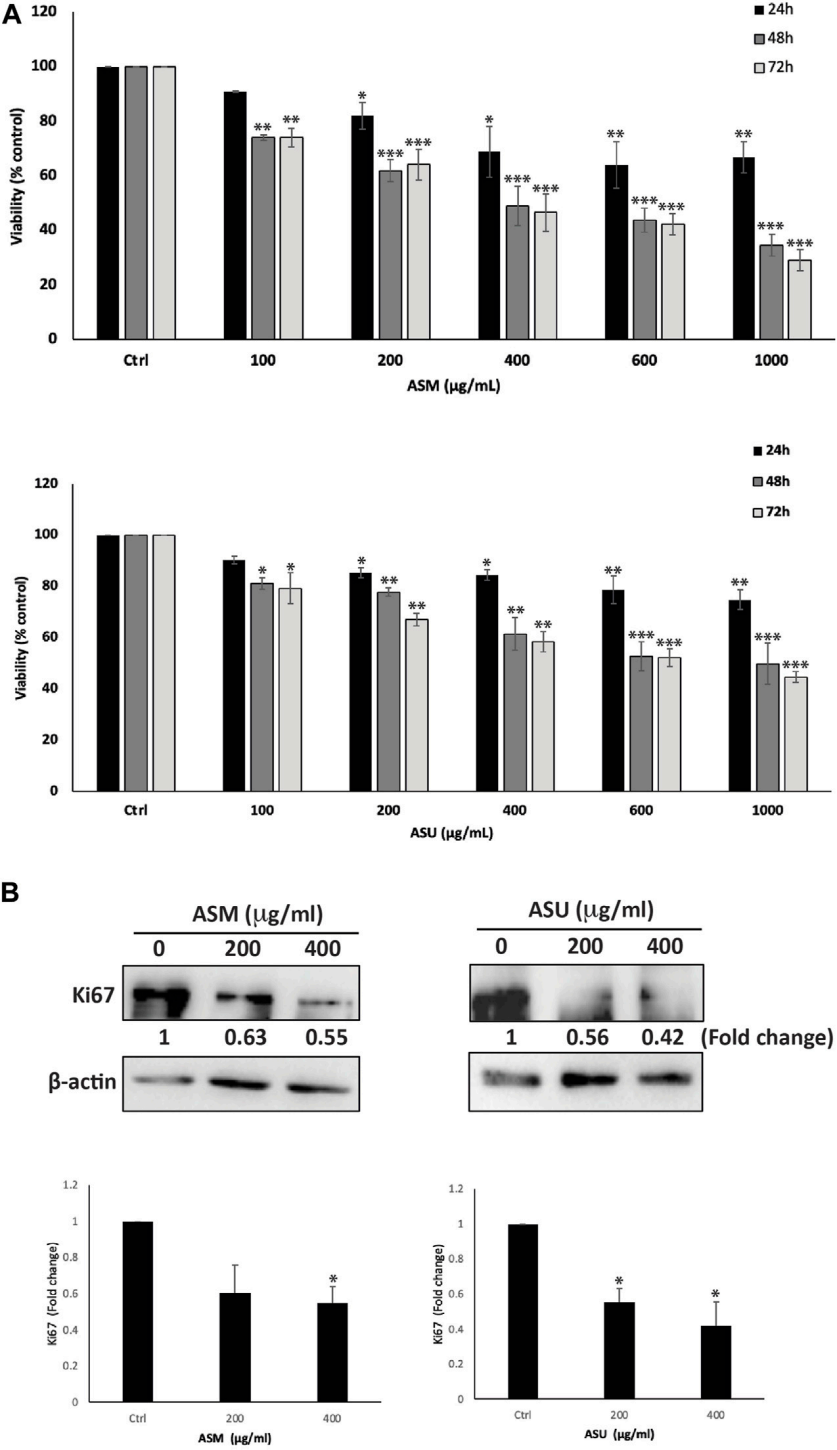
*A. strigosa* extracts inhibit the cellular proliferation of Capan-2 pancreatic cancer cells. **(A)** Capan-2 cells were treated with and without the indicated concentrations of ASM and ASU for 24, 48, and 72 h. Cell viability was examined using the metabolism-based MTT assay, as described in Materials and Methods. Data represent the mean ± SEM of three independent experiments (*n* = 3) performed in triplicate and expressed as a percentage of the corresponding control cells. **(B)** Capan-2 cells were incubated for 24 h with and without the indicated concentrations of ASM and ASU. Cells were then lysed, and protein lysates were subject to Western blotting with a Ki67 antibody, using β-actin as the loading control. Data represent the mean ± SEM of three independent experiments (*n* = 3) and are expressed as a percentage of the corresponding control cells. Statistical analysis was performed using one-way ANOVA followed by LSD *post hoc* test (*p < 0.05, **p < 0.005, ***p < 0.001).

To investigate the anti-proliferative effect of ASM and ASU extracts on pancreatic cancer, the effect of various concentrations of the extracts (0, 100, 200, 400, 600, and 1,000 μg/mL) was tested on the human pancreatic ductal adenocarcinoma Capan-2 cell line at 24, 48, and 72 h of treatment. Results show that ASM and ASU treatments both decreased cell viability in a concentration- and time-dependent manner, with ASM exhibiting stronger activity (Figure 4A). For example, at 48 h of treatment, cell viability using 100, 200, 400, 600 μg/mL of ASM to treat Capan-2 cells was 74% ± 1.1%, 62% ± 4.0%, 49% ± 7.2%, and 44% ± 4.4% that of control cells compared to 81% ± 2.2%, 78% ± 1.6%, 61% ± 6.2, and 53% ± 5.7% when ASU was used. The half-maximal inhibitory concentration (IC_50_) was 2136.6, 404.98, and 370.6 μg/mL at 24, 48, and 72 h, respectively with ASM and 2994.97, 847.92, and 687.46 μg/mL at 24, 48, and 72 h, respectively with ASU. Based on these values, 200 and 400 μg/mL ASM and ASU were used in further experiments.

To confirm the anti-proliferative effect of ASM and ASU on a molecular level, protein lysates from Capan-2-treated cells were immunoblotted with an anti-Ki67 antibody, which is a widely used biomarker that reflects the cell proliferation state and indicates the prognosis for many cancers, including pancreatic cancer (Lee et al., 2013). Figure 4B shows that treatment of Capan-2 cells with 200 and 400 μg/mL of ASM and ASU caused a significant decrease in the levels of Ki67 in a concentration-dependent manner, 24 h after treatment. These data suggest that ASM and ASU inhibit the growth of Capan-2 cells by interfering with their cell-proliferation process.

### 3.5 ASM and ASU induce intrinsic apoptosis in Capan-2 cells

**FIGURE 5:**
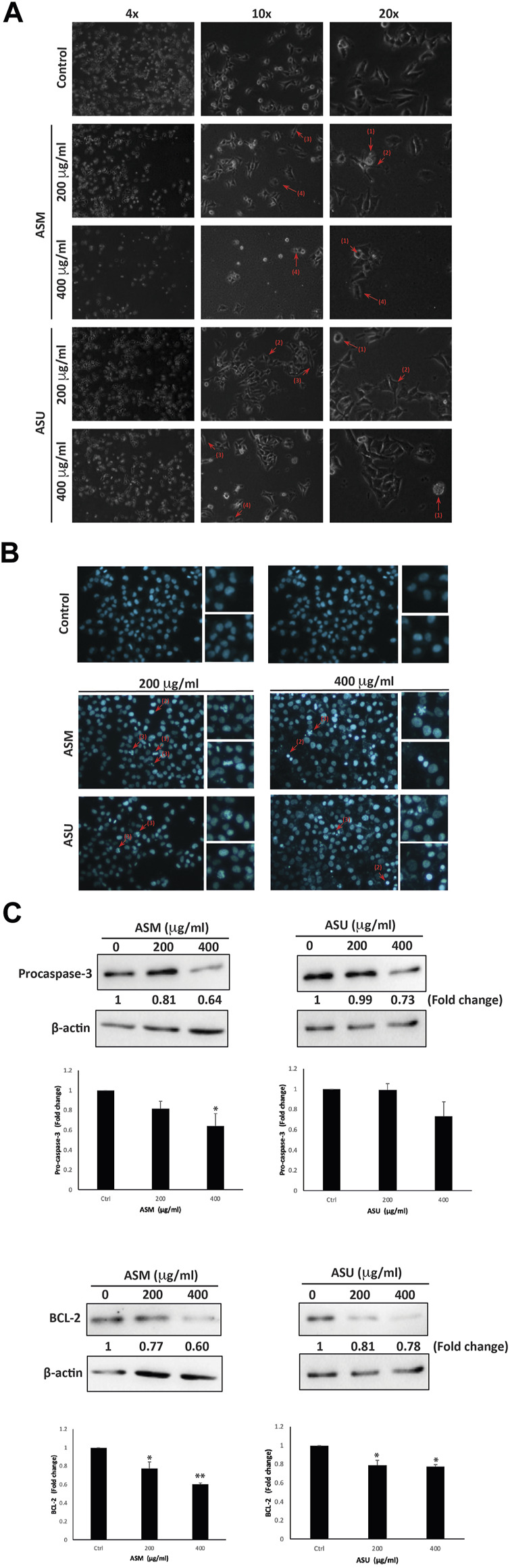
*A. strigosa* induces apoptosis in Capan-2 cells. **(A)** Capan-2 cells were treated with and without the indicated concentrations of ASM and ASU. Morphological changes were observed by light microscopy. Arrows show (1) apoptotic bodies, (2) echinoid spikes, (3) shrinkage and (4) membrane blebbing. **(B)** Cells were incubated with the corresponding concentrations of ASM and ASU for 24 h and stained with 4′,6-diamidino-2-phenylindole (DAPI) to visualize nuclear alterations characteristic of apoptotic features such as (1) chromatin lysis, (2) nuclear condensation, and (3) aggregation of apoptotic bodies. **(C)** Cells were incubated with and without the indicated concentrations of ASM and ASU for 24 h. Protein levels of procaspase-3 and BCL-2 were determined by Western blotting, using β-actin as loading control. Control images are re-used for illustrative purposes. Data represent the mean of ±SEM of three independent experiments (n = 3; *p < 0.05, and **p < 0.01).

At 24 h after treatment with ASM and ASU, Capan-2 cells were examined using an inverted phase-contrast microscope. Results showed a concentration-dependent decrease in the total number of cells per microscopic field. Further analysis of the morphology of the treated and DAPI-stained cells revealed apoptotic characteristics (Figure 5A). Indeed, treated cells clearly showed cytoplasmic shrinkage, rounded morphology, condensation of the nuclear material, the appearance of membrane blebbing, and apoptotic bodies, suggesting the possible induction of apoptosis in Capan-2 cells by ASM and ASU treatment.

We next sought to confirm further that apoptosis is activated in ASM- and ASU-treated cells. To this end, we first evaluated the levels of procaspase-3, which has a crucial role in apoptosis. Procaspase-3 is processed and cleaved into the lower-molecular-weight caspase-3, which is required for the efficient execution of the intrinsic apoptosis pathway. Results showed a significant decrease in the levels of procaspase-3 in cells treated with ASM and ASU in a concentration-dependent manner, achieving a significant decrease from the control at 400 μg/mL (Figure 5C). More specifically, ASM had a stronger effect than ASU, with 0.64 ± 0.13- and 0.73 ± 0.14-fold reductions, respectively.

B-cell lymphoma 2 (BCL-2) is an antiapoptotic protein that also plays an important role in the intrinsic apoptosis pathway, and its dysregulation has been linked with the chemoresistance of many cancers by blocking drug-induced apoptosis (Maji et al., 2018). In our study, both ASM and ASU extracts remarkably decreased the level of BCL-2 protein in Capan-2 cells 24 h after treatment. ASM had a greater effect than ASU in a concentration-dependent manner, showing 0.77 ± 0.05- and 0.60 ± 0.02-fold reductions at 200 and 400 μg/mL, respectively, relative to vehicle-treated cells (Figure 5B). Together, these results strongly suggest that *A. strigosa* extracts induce cell death by targeting apoptotic mechanisms and that they could have a potential role in the treatment of pancreatic cancer.

### 3.6 ASM and ASU increase the aggregation of Capan-2 cells

**FIGURE 6:**
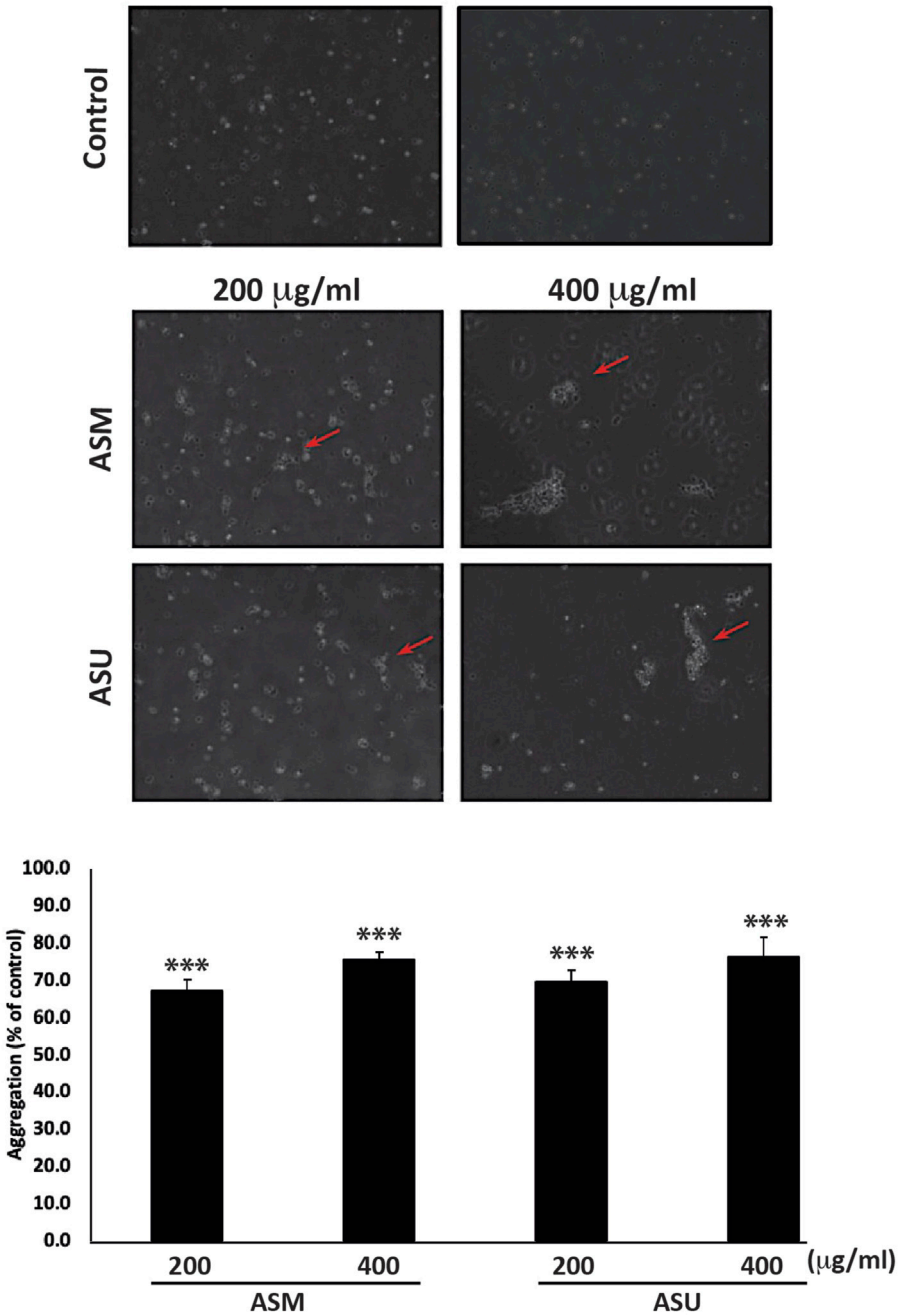
*A. strigosa* extracts increase the cell-cell aggregation of Capan-2 cells. Capan-2 cells were treated with and without the indicated concentrations of ASM and ASU and subjected to an aggregation assay as described in Materials and Methods. Micrographs were taken after 4 h and the percentage of cell-cell aggregates was measured using the equation: % aggregation = (1—Nt/Nc) x 100, where Nt is the number of single cells in the control and Nc is the number of single cells in the treated sample. Data represent the mean ± SEM of three independent experiments (n = 3; *p < 0.05, and **p < 0.005).

Epithelial-mesenchymal transition (EMT) is a complex developmental program that enables cancer cells to infiltrate and metastasize by suppressing their epithelial features, losing their adherence junctions, and transforming into mesenchymal cells to attain mobility. Pancreatic cancer cells, including Capan-2, exhibit an EMT phenotype (Wang et al., 2017). Therefore, blocking or reversing EMT by allowing cells to regain their epithelial properties, such as cell-cell adhesion, could be an effective strategy to improve cancer treatment (Sinha et al., 2020). Toward this end, an aggregation assay was performed to test the effect of *A. strigosa* extracts on the cell-cell adhesion properties of Capan-2 cells in suspension. Figure 6 shows that ASM and ASU caused a concentration-dependent increase in cell-cell aggregates compared to the control cells, with significant 75.8% ± 1.9% and 76.4% ± 5.4% increases, respectively, after 4 h of incubation at 400 μg/mL.

### 3.7 ASM and ASU reduces the migration property of Capan-2 cells

**FIGURE 7:**
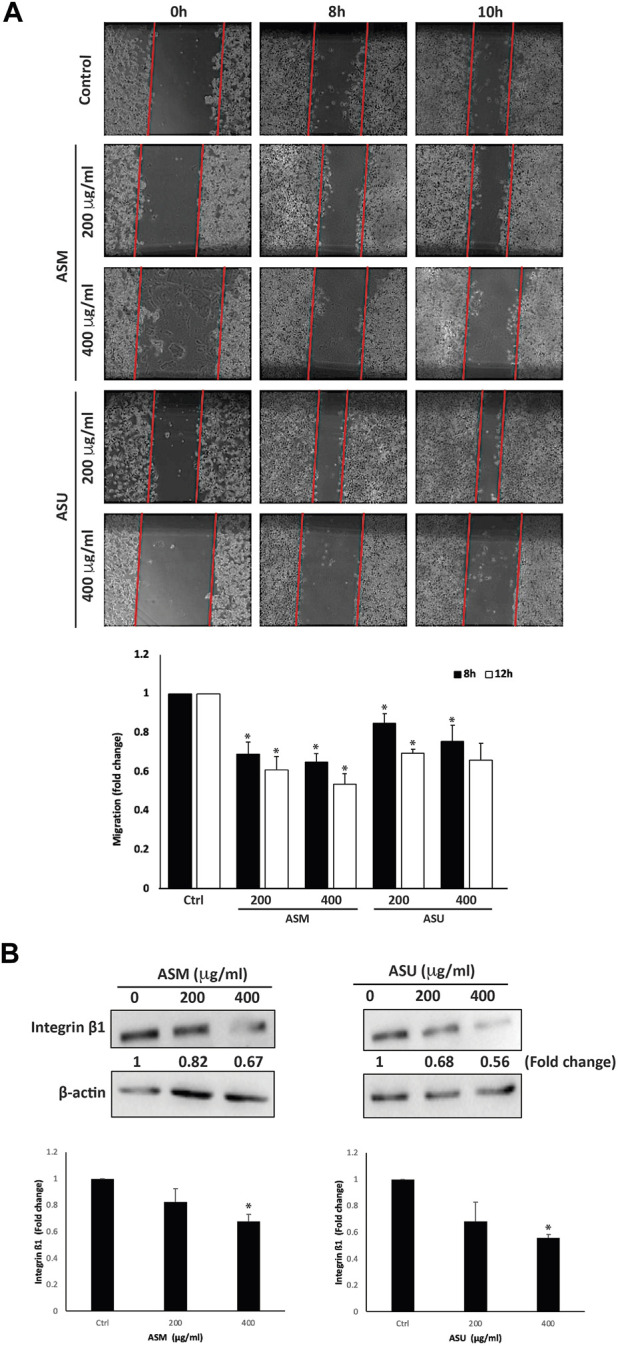
*A. strigosa* extracts inhibit the migration of Capan-2 cells. **(A)** Confluent cultures of Capan-2 cells were wounded by scratching with a pipette tip. The cells were then incubated with and without the indicated concentrations of ASM and ASU. The wound was photographed after 8 and 10 h using an inverted phase-contrast microscope, measured, and analyzed. Values are plotted as fold change compared with the control cells. **(B)** Capan-2 cells were incubated for 24 h with or without the indicated concentrations of ASM and ASU, and whole-cell extracts were subjected to Western blotting analysis for integrin β1 expression using β-actin as the loading control. Control images are re-used for illustrative purposes. Data represent the mean ± SEM of three independent experiments (*p < 0.05).

Cell migration is an essential mechanism during physiological processes such as embryonic morphogenesis, immune responses, and wound repair. It is also a pivotal process contributing to the initial steps of cancer metastasis and a main characteristic of a malignant phenotype. The effect of ASM and ASU extracts on the migration of Capan-2 cells was examined using the wound-healing assay. Figure 7A shows that both *A. strigosa* extracts decreased the Capan-2 cells’ capacity to migrate and fill the wound in a concentration- and time-dependent fashion. For example, 8 h after the scratch was done on the cell monolayer, the distance migrated by Capan-2 cells treated with ASM and ASU at 400 μg/mL was 0.65 ± 0.04- and 0.76 ± 0.08-fold, respectively, that of the control cells.

The spread of a cancer from its primary location to other parts of the body is a multi-step process. Each step requires specific molecular interactions between the tumor cells and the extracellular matrix (ECM) that starts with the adhesion of the cells to the ECM. The cells then secrete hydrolytic enzymes that degrade the matrix, thereby enabling cells to migrate through these modified regions. The adhesion of the cells to the ECM is mediated by the interaction between matrix proteins and specific adhesion molecules, such as integrins, expressed on the cell surface. These allow the formation of focal contacts that drive the cell’s migration process. Overall, integrins play a crucial role in the metastasis of cancers by modulating the interaction of the tumor cells with a wide range of matrix proteins in the ECM (Bozzuto et al., 2010). Studies have shown that the upregulation of integrin subunits is associated with an increase in the metastatic potential of tumor cells, including those of pancreatic adenocarcinoma (Grzesiak et al., 2007). Here, we investigated the levels of integrin β1 in Capan-2 cells after treatment with *A. strigosa* extracts. The results showed that both ASM and ASU at 400 μg/mL significantly decreased the levels of integrin β1 by 0.67 ± 0.05- and 0.56 ± 0.02-fold compared to the control cells, suggesting that *A. strigosa* inhibits the migration capacity of Capan-2 cells by disrupting the integrin-ECM axis (Figure 7B).

### 3.8 ASM inhibits the COX-2 signaling pathway

**FIGURE 8:**
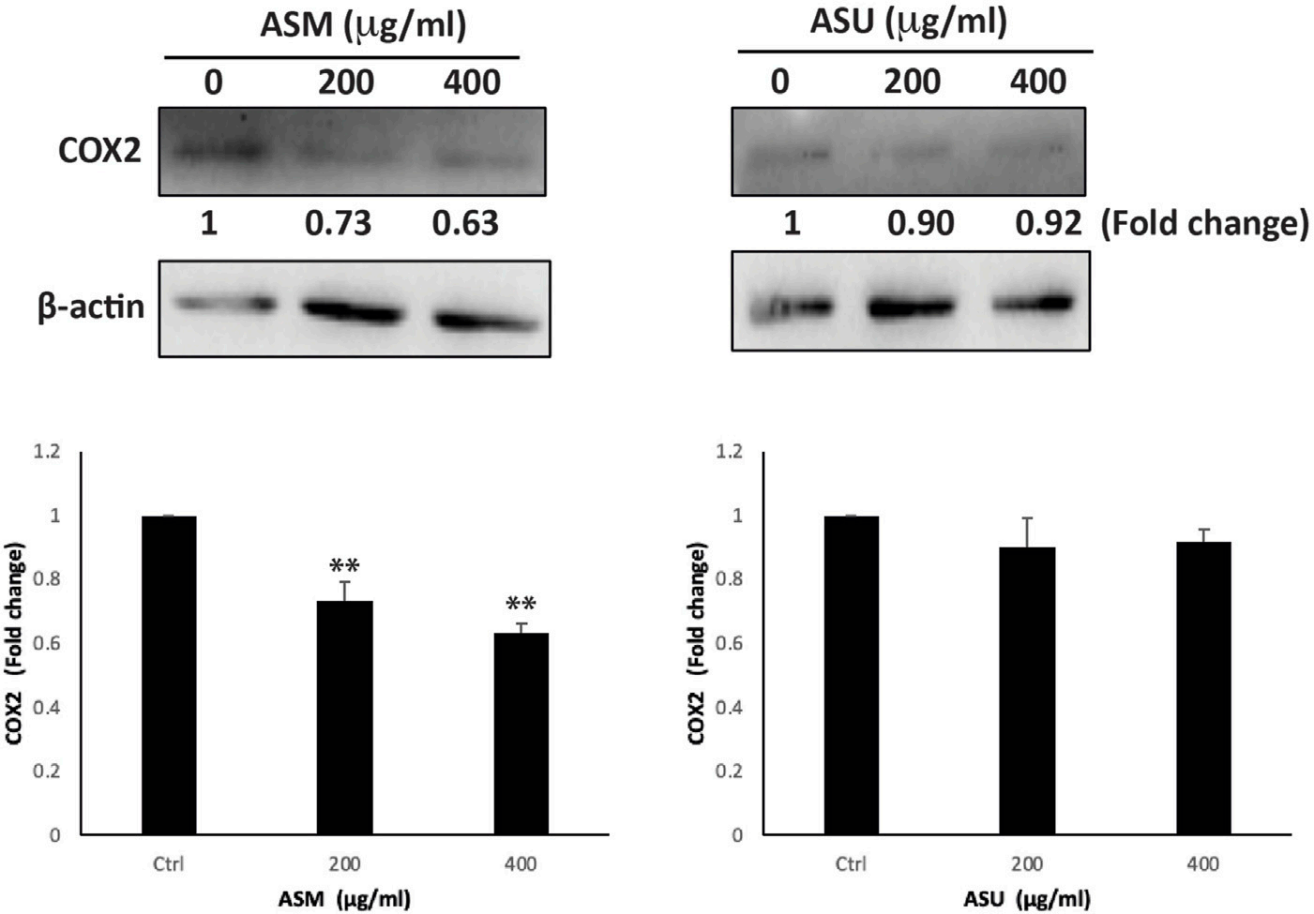
ASM downregulates the COX-2 levels in Capan-2 cells. Capan-2 cells were treated with and without the indicated concentrations of ASM and ASU. After 24 h, protein lysates were examined for the level of COX-2, using β-actin as the loading control. Control images are re-used for illustrative purposes. Data represent the mean ± SEM of three independent experiments (**p < 0.005).

Cyclooxygenase-2 (COX-2), a key enzyme mediating prostaglandin synthesis, has been shown to play a significant role in carcinogenesis. Studies have shown that COX-2 is overexpressed in most cancers, including pancreatic ductal adenocarcinoma, and, that is, associated with poor prognosis (Hill et al., 2012). In fact, it has been linked with tumor development, proliferation, progression, and metastasis, in addition to chemoresistance. Moreover, the inhibition of COX-2 has been shown to reduce the risk of metastasis and sensitize cancer cells to radio- and chemotherapy. Therefore targeting the COX-2 pathway has a promising therapeutic potential, paving the way for the development of new anticancer drugs (Mohsin et al., 2022). Our results showed that ASM treatment at 200 and 400 μg/mL caused a significant decrease in COX-2 levels by 0.73 ± 0.06- and 0.63 ± 0.03-fold, respectively, compared to the control (Figure 8). However, no significant effect on COX-2 levels was observed in cells treated with the ASU extract, suggesting that ASM potentially contains specific biomolecules implicated in targeting the COX-2 pathway.

## 4 Discussion

In addition to being a reservoir of phytochemicals that protect the plant against environmental constraints and facilitate adaptation, plants are rich in secondary metabolites with varied biological functions that play key roles in the plant’s growth and development (Siciliano et al., 2005; Bhattacharya, 2019). Many of these secondary metabolites are known to possess therapeutic properties with strong antioxidant, anti-inflammatory, and anticancer effects. Contextually, the use of plants and herbal extracts in the treatment of cancer, either alone or in combination with established chemotherapeutic agents, has been increasingly drawing attention. More specifically, several experimental and clinical studies using herbal extracts have shown beneficial effects in pancreatic cancer with low side-effects, suggesting that natural products will play a pivotal role in the identification of novel treatments for this aggressive cancer (Kim et al., 2021; He et al., 2022; Triantafillidis et al., 2022). In the study of medicinal plants, the extraction technique is crucial as it can greatly influence the extract obtained and further the isolation of bioactive compounds (Abubakar and Haque, 2020). In the present study, we investigated the effect of two green extraction methods, maceration, and ultrasound-assisted extraction (UAE), on the composition and antioxidant activity of *A. strigosa*-derived aqueous extracts, as well as their antitumor capabilities against pancreatic cancer. The significance of this study arises from the applicability of the model used, accessibility of the plant, and the cost-effectiveness of the process.

Maceration is a conventional technique that was initially used in wine making and has been widely adopted in plant medicinal research (Azwanida, 2015). It is considered one of the simplest techniques for the extraction of bioactive compounds, and consists of soaking coarse or powdered dried plant material in solvents such as water, ethanol, methanol, ethyl acetate, acetone, *etc.* Although simple and inexpensive, the maceration technique is time-consuming as it often requires large amounts of solvent, which then needs to be concentrated. However, it is convenient for thermolabile plant material. The more-modern UAE method, also called sonication, uses sound energy at high frequency in the extraction in order to disrupt the plant cell wall and facilitate solvent penetration due to cavitation, thereby improving the release of intracellular compounds into the solvent and increasing the extraction yield. UAE has several advantages: it is simple, time-saving, and requires low amounts of solvent. However, it is often difficult to reproduce, and the ultrasonic energy applied may often produce undesirable changes in the phytochemicals or even degrade them, in addition to producing free radicals during the cavitation phenomenon (Vilkhu et al., 2008; Kentish and Feng, 2014; Ameer et al., 2017). This method requires extraction conditions to be optimized as they play a critical role in the biological quality of the extract.

Indeed, several studies have proven the antioxidant capacity of *A. strigosa* (Al-Khateeb et al., 2019; Ghalib and Kadhim, 2021). This plant was also shown to possess several biological and medicinal properties including antimicrobial (Ali-Shtayeh et al., 1998; Al-Salihi et al., 2007; Al-Salihi et al., 2009; Abdulla and Shihab, 2022), neuroprotective (Tsalkovich et al., 2015), anti-ulcer (Abuereish, 1998; Disi et al., 1998; Abbas et al., 2009), anti-arthritis (Alallan et al., 2018), and anti-hyperglycemic (Bacanlı et al., 2016) properties. Moreover, its tumorigenic activity has been recently investigated in a study using root and leaf extracts, highlighting that the latter had a more potent anti-proliferative effect against human breast and colorectal cancer cell lines (Al-Khatib et al., 2021). However, the effect of *A. strigosa* on the malignant phenotype of pancreatic cancer has not been studied to date. Overall, our results show that *A. strigosa* as a crude extract has potent *in vitro* anti-pancreatic cancer activities and particularly highlight the ASM extract as a potential source of bioactive compounds with anticancer properties. Uncontrolled proliferation is a major hallmark of cancer and is characterized by the faulty regulation of cell division and cell death. Consequently, we first demonstrated that both ASM and ASU extracts exhibited strong anti-proliferative activity against Capan-2 cells, in a dose-dependent manner. This was concomitant with a decrease in the proliferation marker Ki67, which is highly expressed in pancreatic cancer and closely correlated with tumor severity. Additionally, inducing apoptosis is an important strategy to control excessive cancer cell proliferation. As such, in the development of cancer medication, targeting apoptotic pathways has been of increasing interest. To further understand the anti-proliferative mechanism of the *A. strigosa* extracts, we investigated the induction of apoptosis. This was characterized by an activation of caspase-3 and downregulation of BCL-2, with a more pronounced effect observed by the ASM extract, suggesting that *A. strigosa* potentially induces intrinsic apoptosis in Capan-2 cells.

We also showed that the extracts affected cell-cell adhesion and the migratory potential of Capan-2 cells, which are crucial elements of the epithelial-mesenchymal transition (EMT) and an indication of the cancer’s progression to metastasis. Indeed, the reduction of cell-cell adhesion increases the migratory and invasive capacities of cancer cells. In our study, treatment of Capan-2 cells with ASM and ASU extracts promoted both the formation of cell-cell aggregates and attenuated their migration, indicating that the EMT phenotype had been reversed and metastasis therefore hindered. Moreover, EMT has been increasingly associated with resistance to targeted therapies and chemotherapies (Song and Faber, 2019). In the case of pancreatic cancer, the poor prognosis and dismal 5-year survival rate of only 9% is attributed to the invasive behavior of the disease as well chemoresistance, with EMT being a major contributor to the development of drug resistance (Gaianigo et al., 2017; Hu and Chen, 2021). EMT is a reversible process as cancer cells can still revert to an epithelial phenotype and therefore anti-EMT compounds are considered an attractive target for cancer treatment, with great therapeutic significance. As such, bioactive compounds in both the ASM and ASU extract may be used to suppress the tumorigenicity of pancreatic cancer and its metastatic potential.

Cyclooxygenase-2 (COX-2) is a key enzyme in the biosynthesis of prostaglandins and plays an important role in inflammation. COX-2 and its downstream effector prostaglandin E2 (PGE_2_), a major mediator of inflammation and angiogenesis, are involved in shaping the tumor microenvironment through the modulation of inflammatory mediators, thereby promoting tumor growth and immune escape (Bell et al., 2022). Enhanced levels of PGE_2_ due to the upregulation of COX-2 have been associated with cancer genesis by promoting apoptotic resistance, proliferation, and metastasis of cancer cells. Moreover, elevated COX-2 levels have been associated with resistance of cancer cells to conventional chemotherapy (Pang et al., 2016). Inhibitors of COX-2 could therefore play a unique role in cancer chemotherapy. Indeed, they have been shown to overcome drug resistance in several cancers, including gastric cancer (Xu et al., 2016), head and neck squamous-cell carcinoma (Saito et al., 2021), and breast cancer (Bell et al., 2022). Contextually, COX-2 is upregulated in pancreatic ductal adenocarcinoma and has been shown to play an important role in its proliferation (Hill et al., 2012). Interestingly, ASM treatment caused a significant decrease in COX-2 levels in a dose-dependent manner compared to the ASU-treated pancreatic cancer cells, suggesting that the ASM extract contains bioactive compounds specifically targeting COX-2 signaling and potentially mediating anti-inflammatory responses that confer its enhanced anti-pancreatic cancer activity compared to the ASU extract. In fact, *A. strigosa* is widely used as a herbal medicine for the treatment of rheumatism and arthritis (Ali-Shtayeh et al., 2000; El Beyrouthy et al., 2008; Abu-Rabia, 2015; Alallan et al., 2018). Its anti-inflammatory activity has been studied *in vivo* against Complete Freund’s adjuvant (CFA)-induced arthritis in rats (Alallan et al., 2018) and ethanol-induced gastric ulcers in laboratory animals (Disi et al., 1998; Abbas et al., 2009). However, the mechanism of action behind the anti-inflammatory effect of *A. strigosa* remains unknown.

The LC-MS analysis revealed that there were differences in the composition of the extracts in terms of type and quantity. We have confirmed the presence of secondary metabolites in *A. strigosa* as previously reported in the literature, namely, rosmarinic acid, caffeic acid, quercetin rutinoside, and kaempferol (Braca et al., 2003; Ghalib and Kadhim, 2021; Yarmolinsky et al., 2022). It has been documented that rosmarinic acid, a major phytoconstituent in ASM, exhibits potent anti-cancer effects against pancreatic cancer (Han et al., 2019; Zhou et al., 2022). Other major secondary metabolites of ASM that are known for their anti-cancer effects include syringic acid (Abaza et al., 2013; Periyannan and Veerasamy, 2018; Gheena and Ezhilarasan, 2019), kaempferol (Lee and Kim, 2016; Kim et al., 2018), and salvianolic acid (Katary et al., 2019; Chuang et al., 2020; Teng et al., 2021). The presence of syringic acid, kaempferol and its derivatives, rosmarinic acid, and salvianolic acids in higher amounts in ASM compared to ASU may be responsible for the superior effects observed in ASM. The differences in the phytoconstituents of ASM and ASU might be due to the degradation of phytochemicals as a result of sonication, suggesting that maceration is a more efficient method for the extraction of bioactive compounds from *A. strigosa.* This is in agreement with several studies proving that while advanced methods such as ultrasound- and microwave-assisted extractions provide efficient extracts with a high yield in a much shorter period of time, conventional methods such as maceration are less damaging (El Maaiden et al., 2022). Moreover, the high sound energy applied in ultrasonic-assisted extraction requires close monitoring of the temperature to prevent excessive heating, in addition to producing free radicals, therefore raising the issue of reproducibility (Azwanida, 2015; Abubakar and Haque, 2020).

## 5 Conclusion

The present study provides preliminary data that highlight the potential of *A. strigosa* aqueous extracts as a source of natural antioxidants with potential health benefits and pharmacological applications. In summary, our results show that *A. strigosa* aqueous extracts obtained by both maceration and ultrasound-assisted techniques exhibit strong antioxidant potential, with the ASM extract showing a more potent capacity as observed by its higher total phenolic content and stronger radical-scavenging activity. This correlates with their anti-proliferative and anti-metastatic effects on the aggressive phenotype of Capan-2 cells, suggesting that *A. strigosa* may be a cost-effective and attractive plant for the development of drugs to alleviate pancreatic cancer. Exploring key proteins involved in antioxidant pathways, such as superoxide dismutase (SOD), glutathione peroxidase (GPx), glutathione reductase (GR), and nuclear factor erythroid 2-related factor 2 (Nrf2), is needed to confirm the observed antioxidant profile and to understand the underlying molecular mechanism of action. Moreover, the ASM extract showed a particularly stronger inhibition of the pro-inflammatory mediator COX-2. Overall, our findings provide evidence for the *in vitro* anticancer effect of *A. strigosa* and open new opportunities for investigating its anti-inflammatory effect. While the UAE method provides an effective extract, the conventional maceration method offers an extract with more potent biological properties, proving that maceration is more suitable, leading to minimal changes and not affecting the properties of the extract. It is important to mention that the synergy between the various bioactives in a plant crude extract confers its biological activity. Therefore, characterizing and isolating the bioactive molecules in the ASM and ASU extracts and further analyzing the differences in their composition would be worth investigating. As such, bioassay guided fractionation will be carried out to isolate active compounds from the extracts and study their mechanism of action. Moreover, further *in vivo* studies using animal models of pancreatic cancer are needed to validate the efficacy and safety of the *A. strigosa* extracts.

## Data Availability

The raw data supporting the conclusion of this article will be made available by the authors, without undue reservation.
